# The Impact of the Improved Search Object Detection on the SAR Action Success Probability in Maritime Transport

**DOI:** 10.3390/s20143962

**Published:** 2020-07-16

**Authors:** Zbigniew Burciu, Teresa Abramowicz-Gerigk, Wojciech Przybyl, Ireneusz Plebankiewicz, Adam Januszko

**Affiliations:** 1 Faculty of Navigation, Gdynia Maritime University, 81-225 Gdynia, Poland; z.burciu@wn.umg.edu.pl; 2 Military Institute of Engineer Technology, 50-961 Wroclaw, Poland; przybyl@witi.wroc.pl (W.P.); plebankiewicz@witi.wroc.pl (I.P.); januszko@witi.wroc.pl (A.J.)

**Keywords:** signature, thermal range, radar range, search object, pneumatic life raft, person in the water, SAR action

## Abstract

This paper presents the investigations on the improvement of search object detection during search and rescue (SAR) action at sea using thermal imaging and radar sensors. The introduction of new materials in the construction of lifesaving appliances increasing their detectability has been studied for the selected example of a pneumatic life raft. The research was based on laboratory tests and open sea trials. The presented experimental investigations on the new materials that can be used for pneumatic life raft construction showed the enhancement of its thermal and radar signatures, which directly affect life raft detectability and influence reliability of SAR action and probability of success (POS). The improved detectability of a life raft related to the time to survive of a person in the water (PIW) allowed to present the modified search pattern for both PIW and life raft, significantly increasing POS.

## 1. Introduction

Striving to improve safety in maritime transport, especially after maritime accidents, ship disasters and conducted rescue operations, the legal regulations are created in the field of safety improvement of rescue systems and construction solutions of life saving appliances that aim to eliminate threats related to the loss of life, property and harsh to environment in sea transport. These tasks are implemented by International Maritime Organization (IMO).

According to Annual Overview of Marine Casualties and Incidents 2019, the statistics of the years 2011–2018 give the of 230 ships lost, 23073 casualties & incidents, and 7694 persons injured. Over the 2011–2018 period, 426 accidents led to a total of 696 lost lives, with a very significant decrease since 2015, which was however somewhat reversed in 2018 where 2655 ships in emergency needed Search and Rescue (SAR) services assistance—1225 of them were fishing vessels. Seventy-one percent of SAR operations were related to ship failures and 29% to personal casualties. 

The search and rescue of persons in distress at sea is co-coordinated by local SAR organizations with respect to the international treaties—International Convention for the Safety of Life at Sea (SOLAS) and the SAR Convention of 1979. The guidelines for a common aviation and maritime approach to organizing and providing SAR services are included in the International Aeronautical and Maritime Search and Rescue Manual (IAMSAR) jointly published by International Maritime Organization (IMO) and the International Civil Aviation Organization (ICAO).

The main purpose of the rescue operation at sea is to find and save people in emergency in the shortest possible time [1,2]. A man in emergency or missing floating unit with people on board are defined as the search objects. The technical means at the disposal of the rescue services enable to determine the search area, probability of search object containment in this area, and detection of the search object during a systematic search. The search is planned according to that recommended by IAMSAR search patterns. 

The ability to detect a search object depends on the characteristics of the systems used by the rescuers and the search object signatures in the visible, thermal and radar ranges. The modern SAR systems use the advanced methods of detection in radar ranges [3,4,5,6] and infrared thermal ranges [7]. 

An improvement of the search objects detectability allows to reduce SAR action time by the decrease of search time (T) dependent on the width of the search path (W) and increases the probability of success of the action (POS).

The most frequently used lifesaving appliances on all seagoing vessels are pneumatic life rafts. The minimum requirements for the technical and operational characteristics of a pneumatic life raft are contained in the LSA code [8]. The progress in the construction of life rafts tends towards improvement of their detectability and better protection of survivors against the impact of harsh marine environment and hypothermia [1,9,10]; however, the economic criteria applied by shipowners when purchasing ship equipment results in innovations being slowly introduced to the market, often after the technical and operational requirements contained in the regulations have changed.

The paper presents the innovative solutions in the field of detection, construction and materials of a pneumatic life raft, designed and tested within the project carried out by Lubawa S.A., the life raft manufacturer, together with the subcontractors Gdynia Maritime University and Military Institute of Engineering Technology in Wroclaw [10,11]. In particular, the presented studies concern the improvement of the life raft detectability. Based on the obtained results, the impact of increased life raft detection on the probability of success and reliability of SAR action was analyzed. The impact of the increased life raft detectability was assessed on the basis of applicable recommendations and regulations as well as the results of the research previously conducted at Gdynia Maritime University. The improved detectability of a life raft was related to the time to survive of a man in the water in different temperature ranges. This allowed to present the modified search pattern combined for both person in the water and life raft significantly increasing POS. 

## 2. Materials and Methods

### 2.1. Factors Affecting the Reliability of SAR Actions

**Figure 1:**
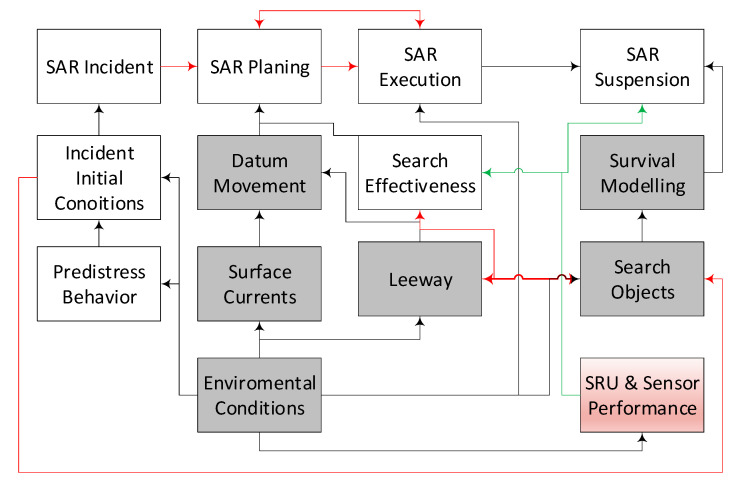
The operating system of rescue operation.

The operating system of the rescue operation (Figure 1) [12] includes both receiving information about the accident, planning and conducting the action, and in the end making decisions about its termination. The grey boxes in Figure 1 indicate the elements of SAR action system affecting the reliability of the action related directly to the search object. 

The most important of them are the characteristics of searched object motions, mainly the speed relative to water, i.e., leeway (wind drift), enabling the determination of its current position to be dependent on the weather conditions and local surface currents [1,12,13,14,15,16].

The reliability of SAR action (1) is a property of the system that indicates whether the action proceeds correctly for the required time (τ_p_), less than the survival time (Sp_t_), in the particular hydro-meteorological conditions, and is ended with finding and saving the rescuers [1,15,17].

(1) $$R_{\mathrm{SAR}}\left(t\right)=P\left({\mathrm{Sp}}_{t}\geq \tau_{p}\right),$$

where: R_SAR_(t) is the reliability of the search and rescue operation system, τ_p_ is SAR operation time without a failure, Sp_t_ is the survival time of search objects (survivors).

Increasing the reliability of the rescue operation can be obtained by reducing the time of the search operation and increasing the survival time mainly due to the use of life-saving appliances. This is related to the construction of life rafts including the provided thermal protection to the survivors, reliability and safety of the life raft in difficult weather conditions, and detectability of the life raft by increasing its thermal and radiolocation signatures.

#### 2.1.1. Life Raft Reliability

According to the LSA Code [7], life rafts should survive at sea in various hydro-meteorological conditions for 30 days. The reliability of the life raft R_tr_(t) is defined as the probability that the time of its safe operation τ_p_ is no less than the design time (τ_30_ = 30 days) of the life raft operation at sea (2) [8]:

(2) $$R_{\mathrm{tr}}\left(t\right)=P\left(\tau_{p}\geq \tau_{30}\right)$$

The main characteristic of the life raft enabling the determination of its most probable position (the reference position P_0_) and size of search area (A_0_) is leeway. The leeway functions of life rafts in relation to wind speed (Figure 3) have been the subject of extensive research [1,10,12,13,14,15,16,18,19] and are used in models for determining the search area.

**Figure 2:**
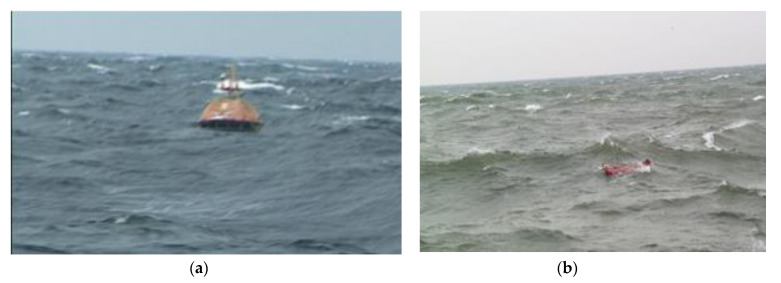
Field experiments of leeway: life raft (**a**) and person in the water (**b**) [20].

**Figure 3:**
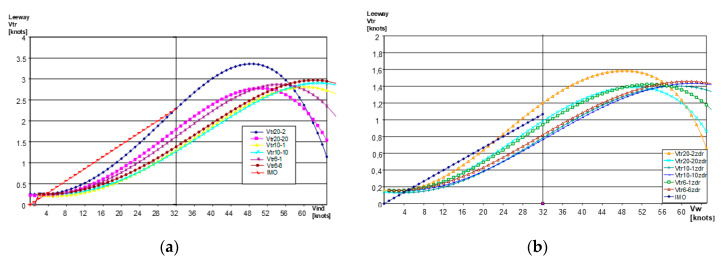
Results of leeway tests of 6-, 10- and 20-person life rafts, occupied by 1 person and by the maximum allowable number of persons, depending on the wind speed, for life rafts without drogues (**a**) and with drogues (**b**) [14].

The research conducted in real sea conditions by Gdynia Maritime University, allowed to determine the leeway functions for PIW and life rafts (Figure 2 and Figure 3) as well as safety function for the life raft dependent on the wind speed and number of survivors on board.

The results of leeway tests for 6-, 10- and 20-person life rafts occupied by 1 person and by the maximum allowable number of people for this life raft, depending on the wind speed, for the life rafts without a drogue (drift anchor stabilizing the life raft motion) and for life rafts with the drogues are presented in Figure 3. 

The life raft safety function developed on the basis of data collected during real scale tests, determines the probability of safe life raft operation within the range of wind speed the life raft is not damaged and has not lost its stability [14]. The loss of stability and overturning under the strong wind means the survivors cannot use it any more. The probability that the maximum wind speed value under which the life raft can be safely operated is no less than the current wind speed can be defined as the life raft safety function (reliability of life raft operation) by Equation (3).

(3) R(x)=P(Z>x),

where R(x) is the reliability of life raft operation, Z is the maximum wind velocity with respect to the life raft safety, and x is the current wind velocity. 

The probability can be determined by formula (4) [14]:

(4) $$P\left(Z>x\right)=1-\int_{0}^{x}f_{z}\left(z\right)\mathrm{dz},\;z>0$$

The function f_z_(z) for the particular life raft is dependent on the current hydro-meteorological conditions and number of the survivors on board the life raft. 

#### 2.1.2. Probability of Survival 

**Figure 4:**
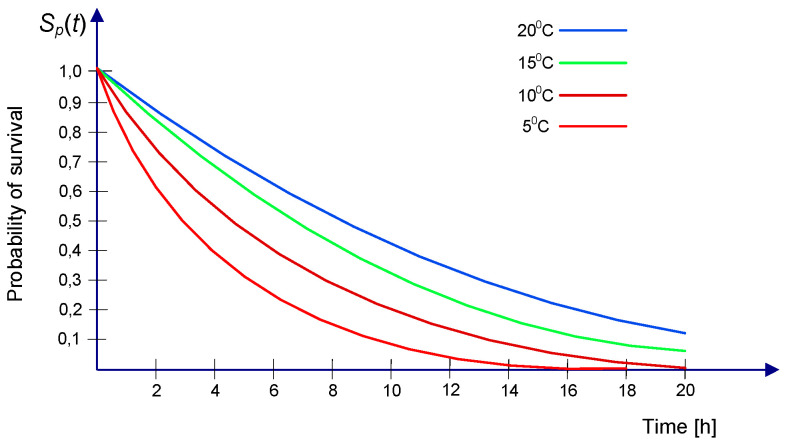
Probability of survival of a person in the water.

The probability of survival of PIW without thermal protection in dependence on the time spent in the water at a given temperature is shown in Figure 4 [21].

**Figure 5:**
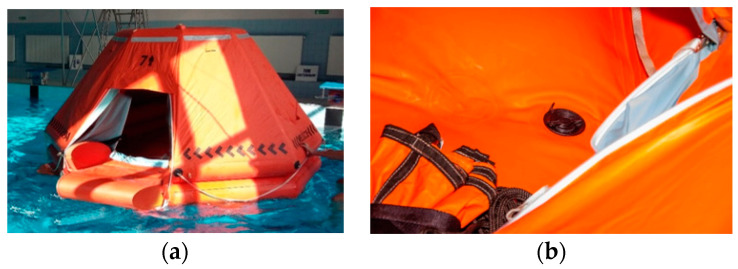
The life raft prototype with the double layered canopy and a ramp to make easier entry from the water into the life raft (**a**), a view of the inner bottom—the floor of the life raft with a valve of the self-draining system (**b**).

The survival time of people in the life raft depends on their protection against the marine environment, mainly proper insulation from external conditions, protection against the body heat loss and hypothermia. The life rafts commonly used on sea-going vessels having the floor and canopy made of a single layer of rubberized or plastic coated fabric can meet the above requirements only partially. The proposed solutions to this problem used in the new construction developed within the project on innovative means of transport in situations of danger to life at sea [10] were the double layer canopy and double bottom with the inner floor automatically drained by a system of return valves eliminating water inside the life raft (Figure 5).

### 2.2. Detectability of the Search Object at Sea

**Figure 6:**
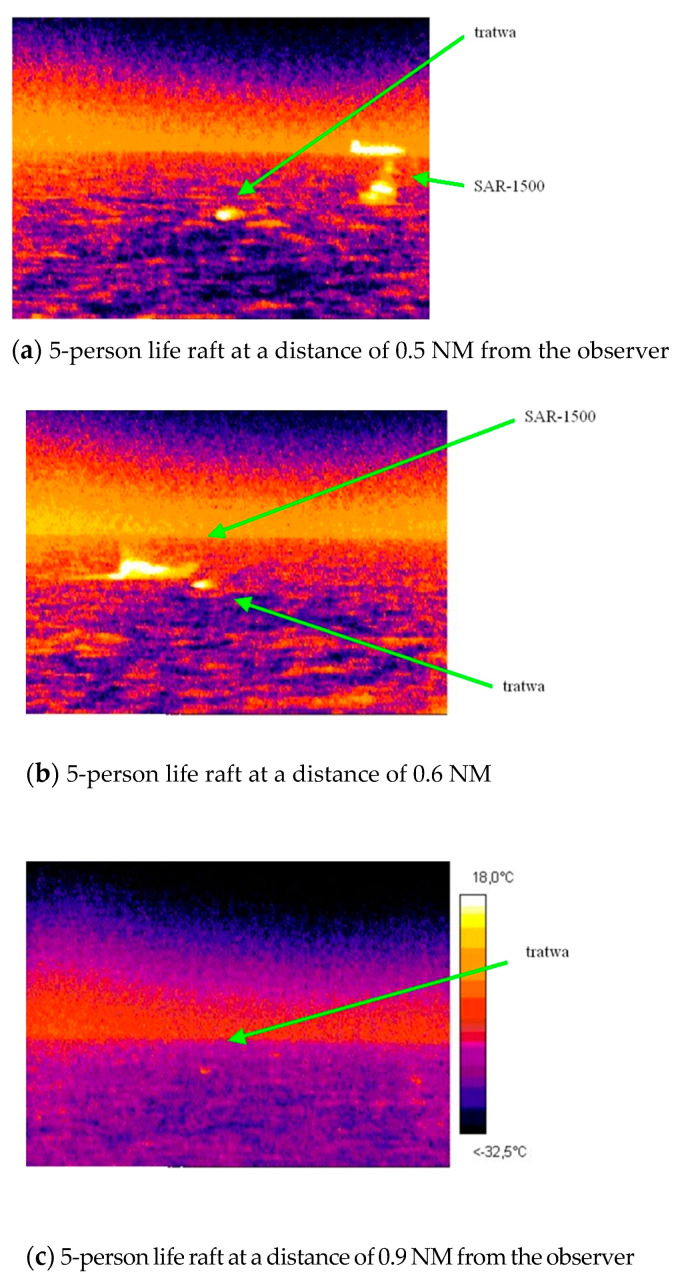
Thermal images of the search area, SAR 1500 rescue vessel and life raft with five persons on board visible at distances of 0.5, 0.6 and 0.9 NM from the observer.

The improved detectability of the search object reduces the time of the search action and increases the action reliability. The tests carried out in the Gulf of Gdansk on board the training vessel Horizon II of Gdynia Maritime University using the thermal imaging camera AGEMA Thermovision 550 allowed to assess the distance from which the life raft can be detected. Figure 6 presents a thermal image of the search area, in which the SAR 1500 rescue vessel and the life raft are visible at the distances of 0.5, 0.6 and 0.9 NM from the observer [20]. 

In the cold waters of the Baltic Sea, where the temperature is 14–20 °C in summer and 2–3 °C in winter, the time to search the survivors is the most important. 

**Figure 7:**
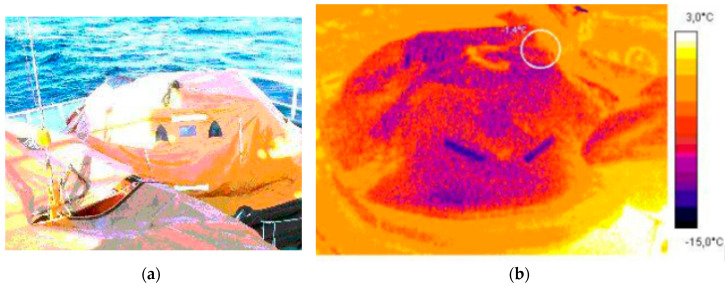
20-person Viking life raft on board training vessel Horizon II (**a**), thermal images of the empty life raft, maximum temperature 1.4 °C (**b**).

**Figure 8:**
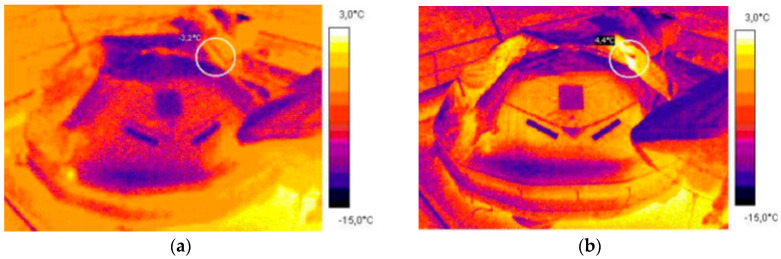
Thermal images of the 20-person Viking life raft: maximum temperature 3.2 °C immediately after 10 men boarding (**a**) and 20 min after their boarding 4.4 °C (**b**).

The trials conducted on board the training vessel Horizon II in winter allowed to identify the thermal signatures of life rafts without survivors and with survivors on board [7]. The thermal images of the empty life raft on board ship before launching, the life raft immediately after boarding by 10 crew members and 20 min after their boarding are presented in Figure 7 and Figure 8 [20].

The observed thermal range of 1.4 °C to 4.4 °C of the tested life raft means that the presence of survivors inside the life raft does not significantly improve the life raft detection. The proposed solution is to use heating elements, increasing the thermal signatures.

#### 2.2.1. Increasing the Signature in the Thermal Range—Laboratory Tests

An improvement of detection efficiency can be accomplished using two spectral cameras (conventional bands: 3−5 μm and 8−14 μm) with very high sensitivities in both the geometric and temperature resolutions, and using specialized image processing techniques, modern algorithms aimed at improving their quality and detection of expected anomalies. The further improvement is possible due to the enhancement of the life raft visibility to thermal imaging cameras via application of heating elements on the surface of life raft canopy, increasing its thermal signature [20].

Three elements were adopted for the analysis of their heating properties:

- heating mats made of nanocomposites based on carbon nanotubes embedded in silicone,
- heating mats made of nanocomposites based on carbon nanotubes coated with fiber on both sides and embedded in silicone,
- thermo-adhesive heating mat made in the form of resistance pathways applied onto an elastic dielectric material.

**Figure 9:**
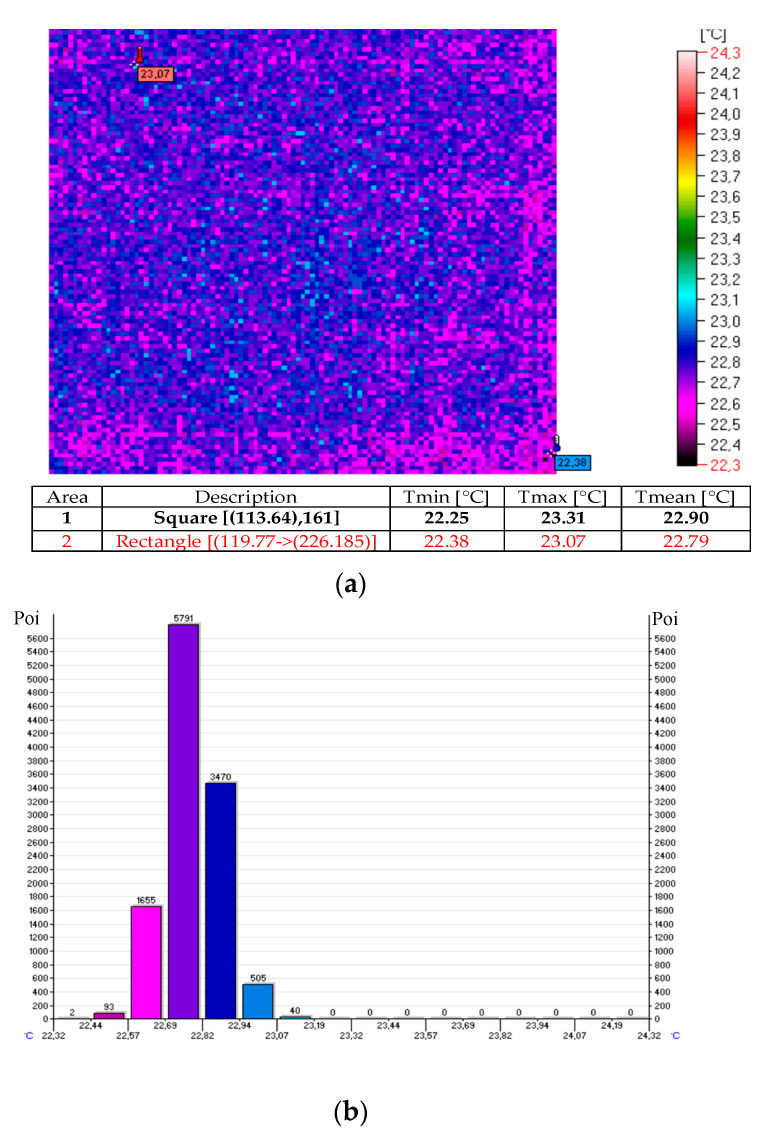
Thermal image (**a**) and histogram (**b**) of the nanocomposite (sample No. 10) at room temperature.

**Figure 10:**
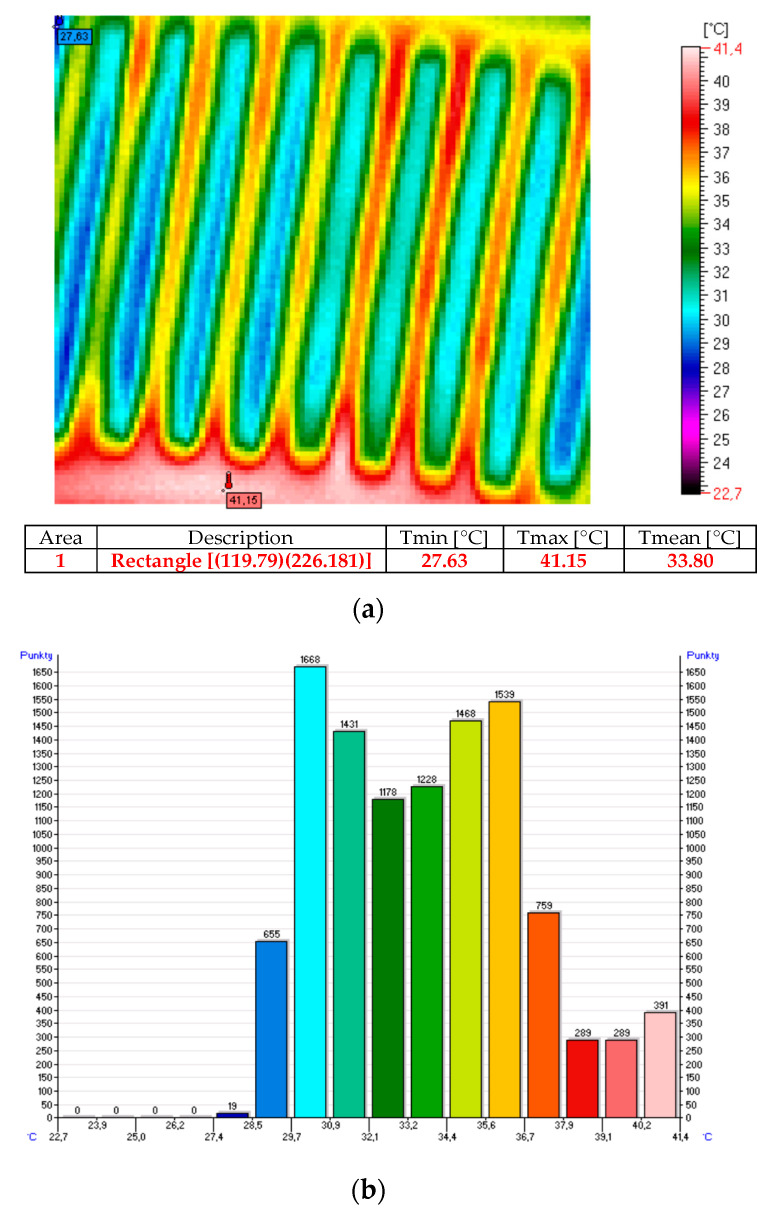
Thermal image (**a**) and histogram (**b**) of the nanocomposite (sample No. 10) following 27 min of the test for 12 V.

**Figure 11:**
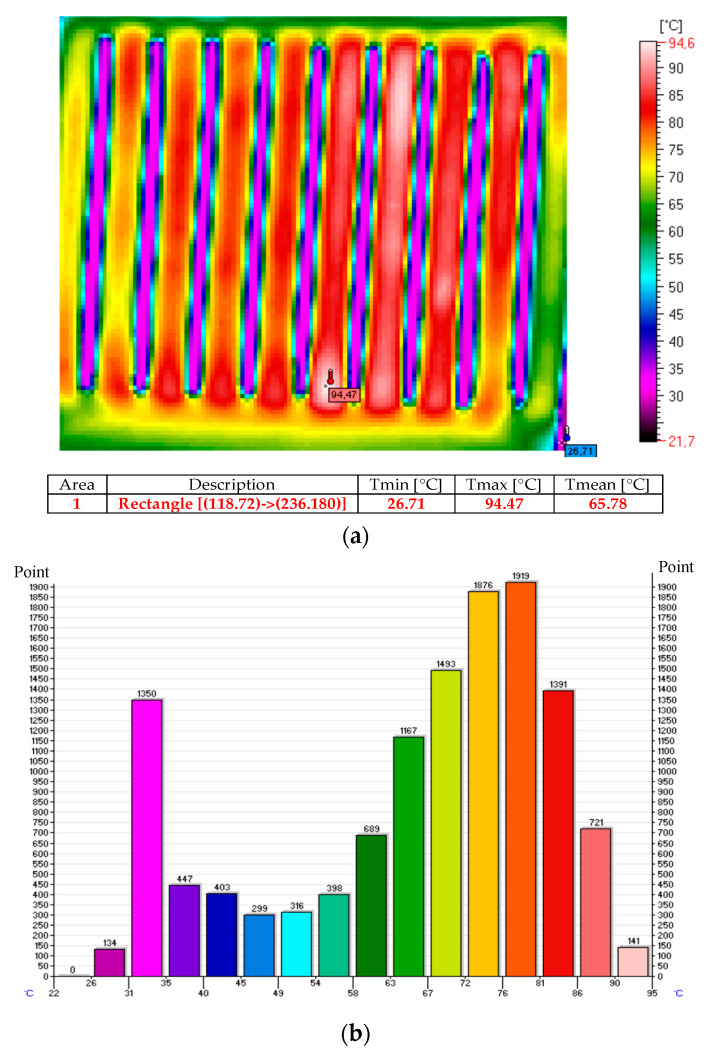
Thermal image (**a**) and histogram (**b**) of the nanocomposite (sample No. 10) following 27 min of the test for 24 V.

The thermal images and histograms of the nanocomposite (sample No. 10) at room temperature following 27 min of the test for 12 V and 24 V are presented in Figure 9, Figure 10 and Figure 11 [11].

**Figure 12:**
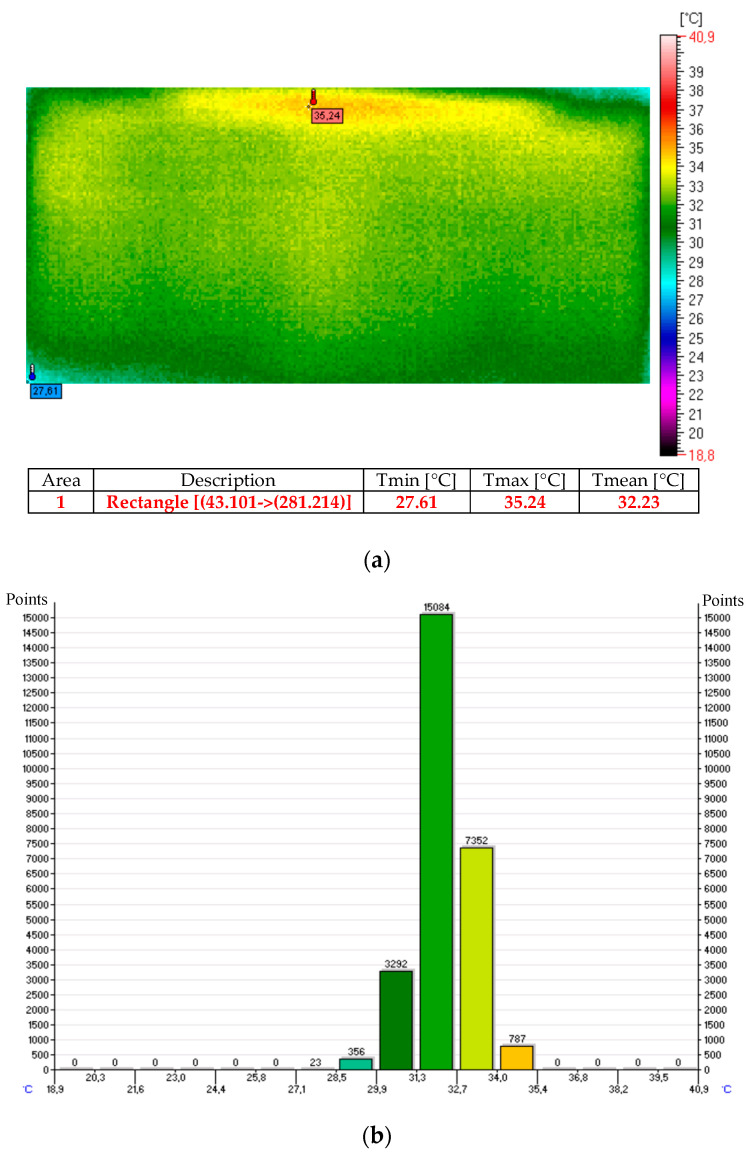
Thermal image (**a**) and histogram (**b**) of the nanocomposite (sample No. 11) following 10 min of the test for 12 V.

**Figure 13:**
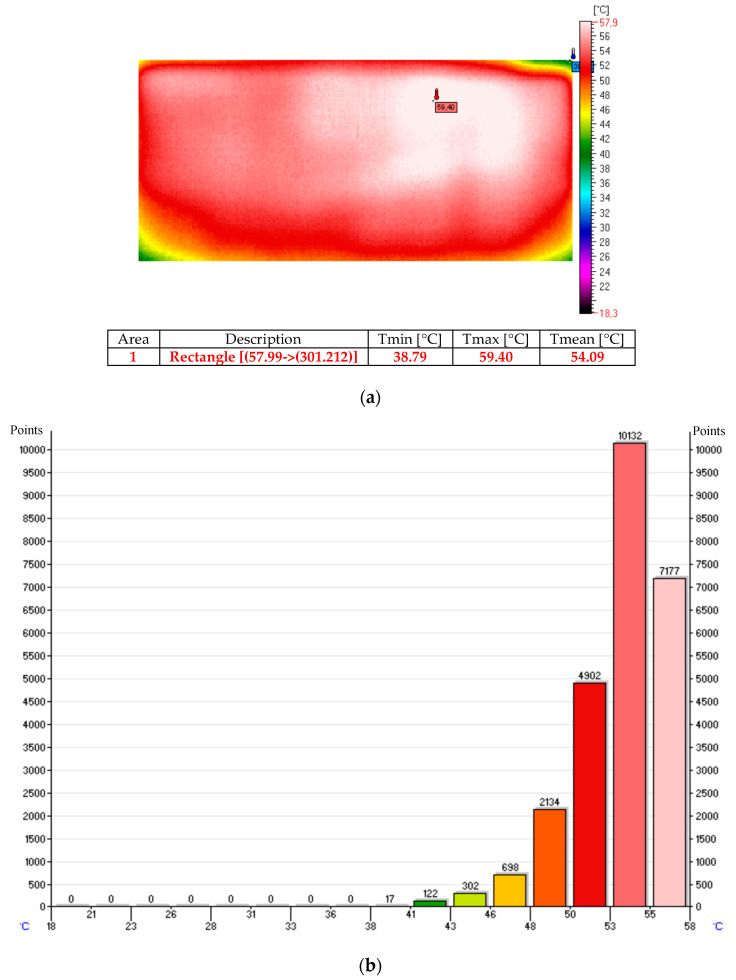
Thermal image (**a**) and histogram (**b**) of the nanocomposite (sample No. 11) following 10 min of the test for 24 V.

The thermal images and histograms of the heating mats made of nanocomposites based on carbon nanotubes coated with fiber on both sides and embedded in silicone following 10 min for the 12 V and 24 V tests are presented in Figure 12 and Figure 13 [11].

**Figure 14:**
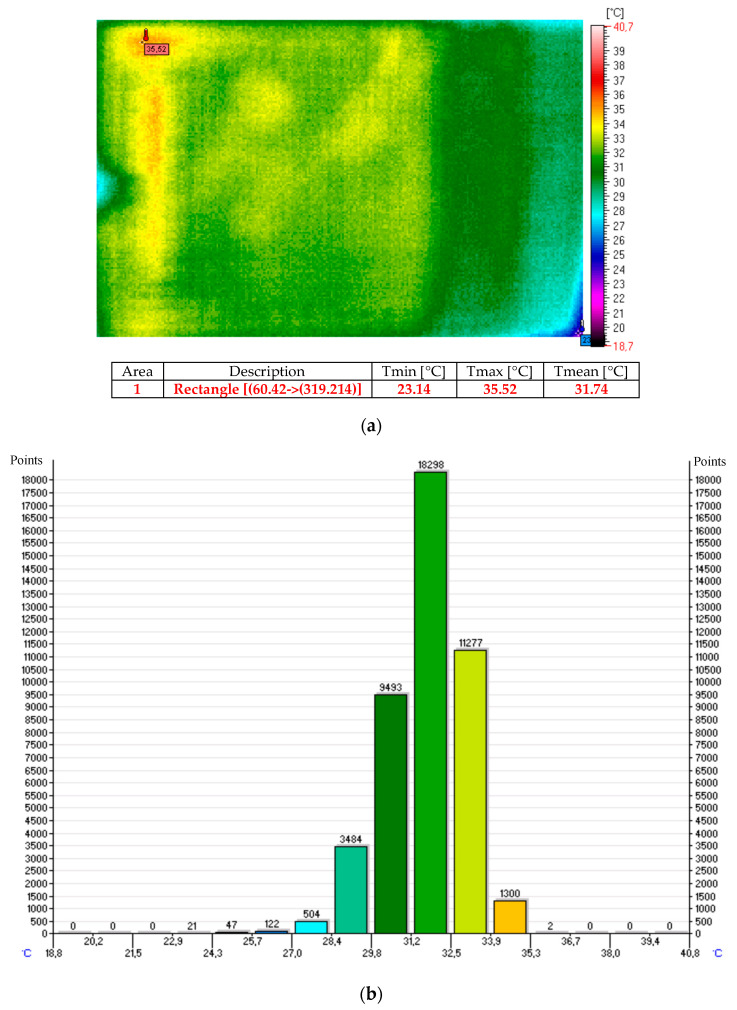
Thermal image (**a**) and histogram (**b**) of the heating mat (sample No. T1) following 10 min of the test for 12 V.

The thermal image and histogram of the thermo-adhesive heating mat made in the form of resistance pathways applied onto an elastic dielectric material (sample No. T1) following 10 min test for the 12 V is presented in Figure 14 [11].

The temperature of the mat and in principle the temperature difference in relation to the background is an essential parameter when searching survivors using thermal imagery. 

The results of the presented tests show that with 12 V power supply a similar average temperature was obtained in all tested mats in the temperature range from 31 °C to 33 °C. 

#### 2.2.2. Increasing the Signature in the Thermal Range—Field Experiments

The field experiments were conducted on the pneumatic life raft canopy of the 10-person life raft Stomil Grudziadz PTR 10.

For recording the thermal image, a Testo 890 thermal imaging camera with a resolution of 640 × 480 pixels with a lens with a field of view 15° × 11°, geometric resolution 0.42 mrad and sensitivity <42 mK at 30 °C was used. This means that even the smallest or very distant objects were recorded with an extremely high level of accuracy. 

The entire thermal process was recorded in real time. All data from the thermographic recording was transferred via USB 2.0 to the computer and analyzed. During the measurement, a precise analysis of thermal changes on the surface of the tested objects was carried out and a histogram of the temperature distribution was determined for the entire thermal image. 

**Figure 15:**
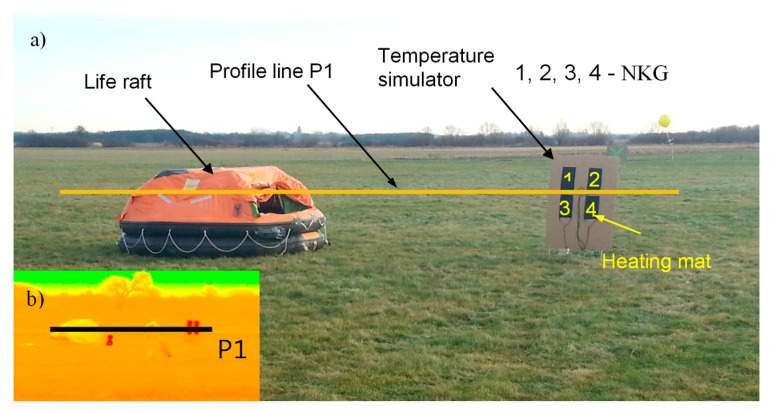
General view of the life raft and temperature simulator: (**a**) visible bandwidth; (**b**) infrared range: 8 ÷ 14 μm band; P1—temperature profile line.

In order to determine the maximum temperatures on individual objects: life raft and temperature simulator a temperature profile line was drawn (Figure 15).

**Figure 16:**
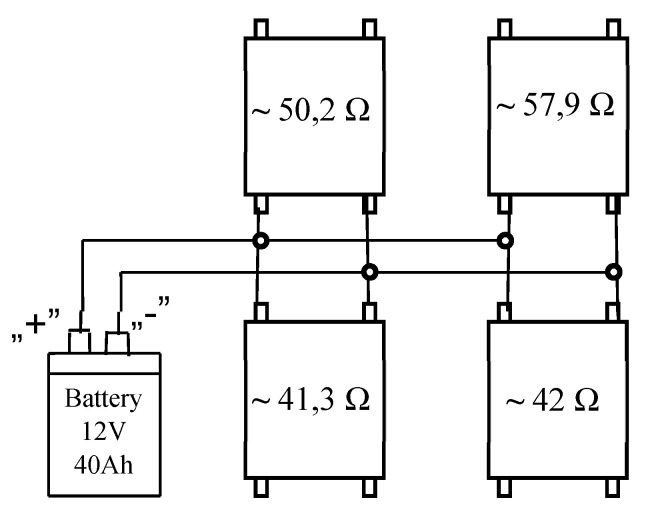
Temperature simulator. Connection method of heating nanocomposite (NKG).

The temperature simulator (Figure 16) was made of insulating material with heating mats made of nanocomposite materials with heating properties.

Heating nanocomposite (NKG) consists of a base fabric with a layer of polymer matrix containing carbon nanotubes. To connect the power supply, the power strips (electrodes) were embedded in the nanocomposite and their ends led outside.

The distribution of mats was adjusted to the life raft shape and their surface covered about 1/4 of the visible surface of the life raft. This surface was the smallest surface measurable by the Testo 890 thermal camera with a 15° × 11° field of view lens from a distance of about 1000 m.

Based on the results obtained from laboratory tests, it was determined that the optimal supply voltage of the heating nanocomposite (NKG) is between 11 V and 16 V; therefore, in field conditions a LiFePO4 12 V 40 Ah battery was used as the power source.

The method of connecting individual mats is shown in Figure 16. 

Despite the disadvantages of the uneven surface temperature distribution observed during the laboratory tests, this method is rational from the point of view of use in the finished product due to the power balance.

**Figure 17:**
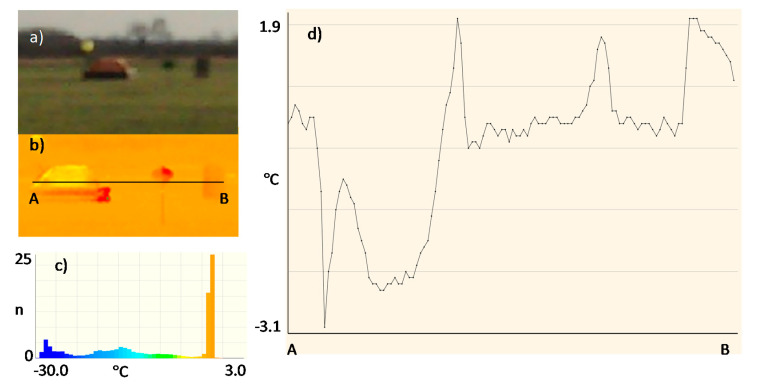
Image of the life raft in the infrared range. Measurement No. 3: (**a**) visible range; (**b**) infrared range: 8 ÷ 14 μm; (**c**) histogram, n—number of pixels; (**d**) temperature profile line.

**Figure 18:**
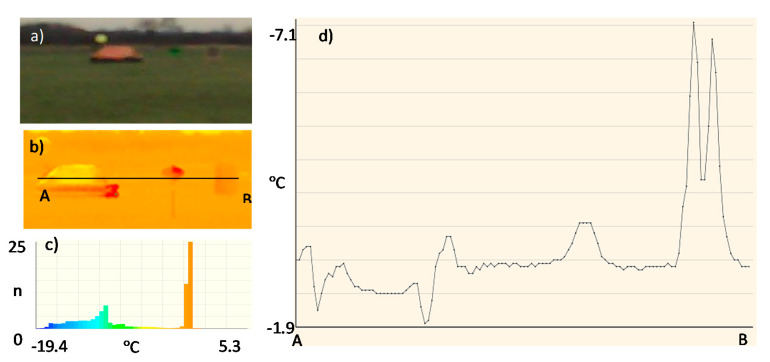
Image of the life raft covered with a sample of metallized fabric in orange. Measurement No. 4: (**a**) visible range; (**b**) infrared range: 8 ÷ 14 μm; (**c**) histogram, n—number of pixels; (**d**) temperature profile line.

**Figure 19:**
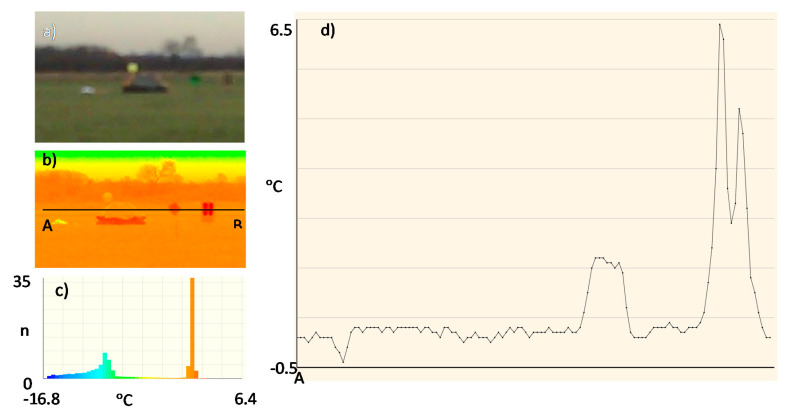
Image of the life raft covered with a sample of metallized fabric in green. Measurement No. 5: (**a**) visible range; (**b**) infrared range: 8 ÷ 14 μm; (**c**) histogram, n—number of pixels; (**d**) temperature profile line.

**Figure 20:**
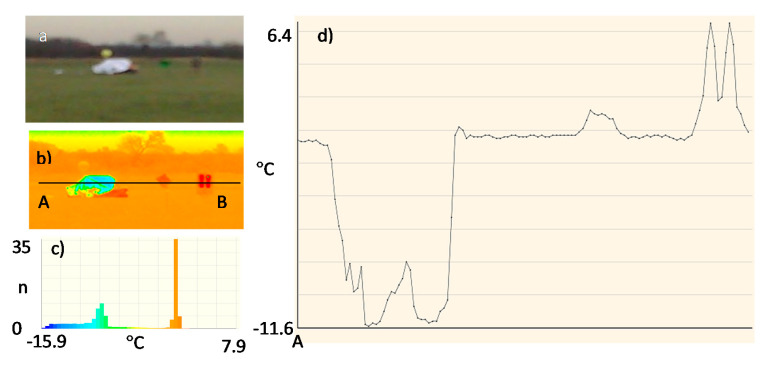
Image of the life raft covered with a sample of metallized fabric ST55-110/21, metallized side of fabric outside, Measurement No. 6: (**a**) visible range; (**b**) infrared range: 8 ÷ 14 μm; (**c**) histogram, n—number of pixels; (**d**) temperature profile line.

**Figure 21:**
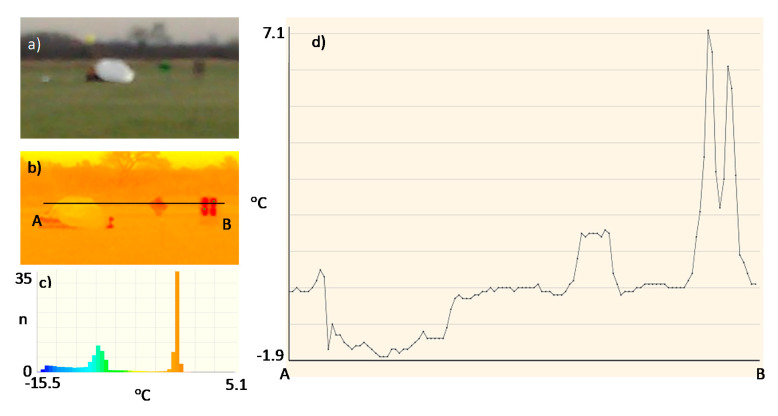
Image of the life raft covered with a sample of metallized fabric ST55-110/21, metallized side of fabric inside, Measurement No. 7: (**a**) visible range; (**b**) infrared range: 8 ÷ 14 μm; (**c**) histogram, n—number of pixels; (**d**) temperature profile.

Due to the assumed use of metallized fabrics to improve detection in the radiolocation range, the measurements were also made for a life raft not covered (Figure 17) and covered with these fabrics:

- metallized fabric provided by Lubawa branch in Grudziadz—orange (Figure 18);
- metallized fabric supplied by Lubawa branch in Grudziadz—green (Figure 19);
- technical one-sided metallized fabric ST55-110/21 made by IZO-TERM Gryfow (Figure 20 and Figure 21).

The thermal imaging was analyzed and processed to determine the temperature differences related to the background and make a selection of the best presentation with respect to the quick identification of the object.

The normalized thermal imaging (after removing noise), containing information about objects with a temperature greater than the background by ∆t = + 5 °C, was adopted. 

The matrix (R+G+B)/3 was used as the initial image. The average value (background) is calculated using formula (5):

(5) $$t_{\mathrm{sr}}=\frac{1}{n}\sum_{i=0}^{n}t_{i},$$

where: t_sr_—average temperature; n—number of elements in the matrix.

**Figure 22:**
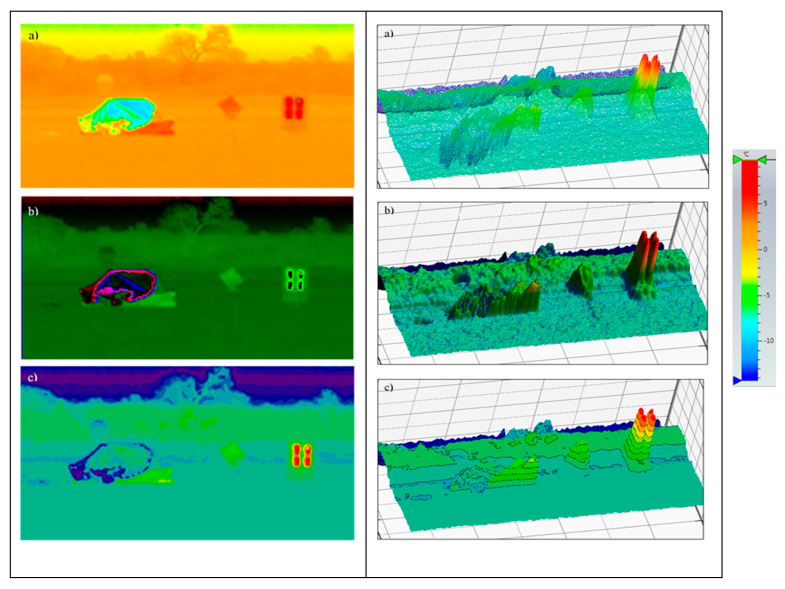
Imaging in the infrared range (left): 8 ÷ 14 μm, (**a**) image recorded by the camera—RGB matrix; (**b**) RGB matrix inversion; (**c**) matrix in the form (R+G+B)/3. 3D image (right): (**a**) represented by means of a color palette; (**b**) represented by a color palette with contour filling; (**c**) represented by a color palette with contour filling and contour lines.

The adopted upper limit Δt = +5 °C is the result of the analysis of the profile lines in Figure 17d, Figure 18d, Figure 19d, Figure 20d and Figure 21d and results from the adopted minimum temperature value for the heating mat turned on (Figure 22).

**Figure 23:**
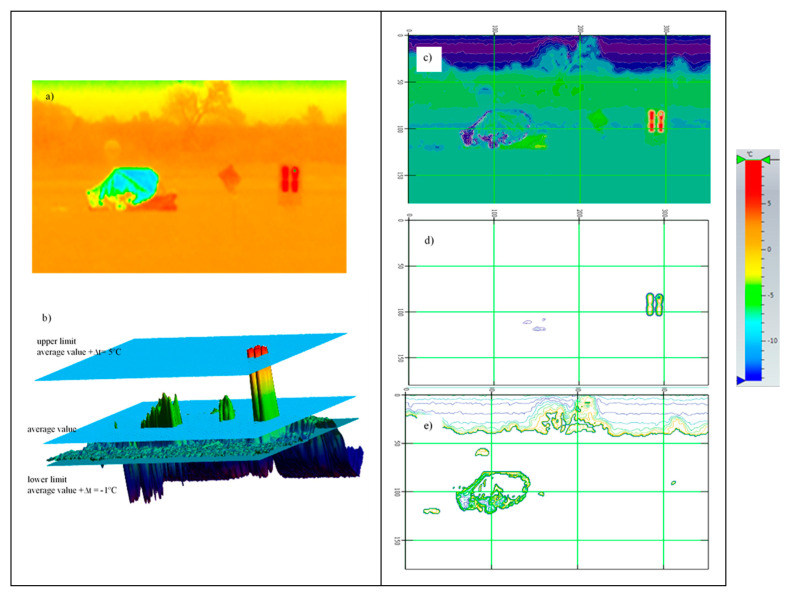
Imaging in the infrared range 8 ÷ 14 μm: (**a**) represented by means of a color palette; (**b**) 3D image represented by a color palette with contour filling with average value, lower and upper limits marked; (**c**) RGB matrix inversion of a) imaging; (**d**) ∆t = 5 °C related to the average value; (**e**) ∆t = −1 °C related to the average value.

The same principle was used for the lower limit Δt = −1 °C. The lower values of the temperature (possible in winter) relative to water of the life raft with a metallized fabric coating with the metallized side inward were taken into account (Figure 23). 

The presented analysis of the thermal imaging in the infrared range 8 ÷ 14 μm shows that the use of heating mats based on nanocomposites can improve the detection of the life raft. Heating elements are clearly visible on the life raft canopy shell and surroundings background enabling its easier detection using thermal imaging sensors. Depending on the weather conditions and sensitivity of the IR sensor, the detection distance of the searched object on the water equipped with heating elements may increase up to three times compared to an object without heating elements.

The use of inside layer of the life raft canopy made of metallized fabric with the inward metallized coating also improves the detection of the raft in the thermal range.

#### 2.2.3. Increasing the Signature in the Radar Range—Laboratory Tests

Increasing the probability of detecting a life raft in the radar range can be obtained by:

- the use of a radar target enhancer (radar reflector) mounted directly on the life raft canopy or on a small mast,
- giving the outer shell of the raft the properties, which would result in radar signals being reflected.

Radar target enhancers in their many forms are currently used on life rafts [21,22,23], however, their efficiency is limited due to their low location on the raft and sea waves interference. Therefore, the proposed solution was to provide the shell of the life raft canopy reflecting radar signals [10,11]. It was decided on purpose to avoid using a corner reflector as a target.

The shell reflecting radar signals can be fabricated of metalized materials in the form of fabric covered with metal foil or by introduction of metallic particles into the rubber layer and applying this enhanced rubber onto the shells. 

**Figure 24:**
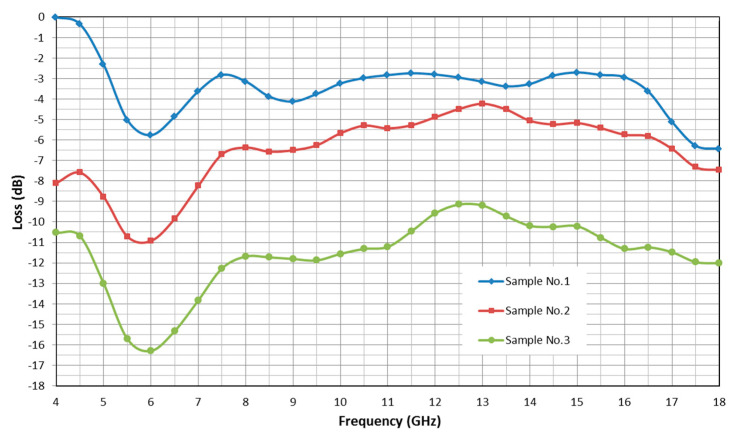
Amplification of the electromagnetic wave by sample No. 1., sample No. 2., and sample No. 3.

The main goal of the tests presented in the paper was to find out the acceptable solution specifically for the fabric Company. The three samples of the materials used for the canopy construction e.g., metallized fabrics provided by “Lubawa” S.A. (“Lubawa” S.A. - Grudziadz branch)—orange (Figure 18)—sample No. 1, green (Figure 19)—sample No. 2, and technical one sided metallized fabric ST55-110/21 produced by “IZO-TERM” Ltd. Gryfow—sample No. 3 (Figure 20 and Figure 21) were tested in the Research Laboratory of Military Institute of Engineer Technology. The samples were flat, square pieces of materials with dimensions of 0.3 m × 0.3 m. The PR-17 reflectometer operating in the range of 4–18 GHz with fixed geometry, keeping the angel between the acting and reflected beams less or equal to 5 degrees (almost perpendicular to the sample) was used for the measurement of the relative amplification of electromagnetic wave. The amplifications of the electromagnetic wave by the samples are presented in Figure 24 [11].

The conducted research showed that most radar radiation energy was reflected by metallized on one side technical fabric No. 1: ST55-110/21—metallized polyester film laminated to fabric. A good result was also obtained for sample No. 2. After the verification of these materials on the life raft at sea, it was proposed to use them in a new life raft prototype [10,11]. 

It was decided to offer the final solution based only on the laboratory results. The geometry of the life raft in conducting rescue operations on the calm sea using radar has an impact on the detection efficiency, however, it can be assumed that both the life raft and sensor placed on the ship will change their mutual position. Thus, not only RCS measurements relative to the read geometry and material properties would affect the object detection performance, but rather it would depend on the probability of being in a mutual position of the sensor and the object, giving maximum RCS. 

## 3. Discussion

### 3.1. Increase of POS by Widening the Sweep Width of a Search Pattern

The increase of thermal and radar signatures increasing the range of a search object detectability in SAR action allows to widen the sweep width W of a search pattern in the determined search area and decrease the time to search T. 

**Figure 25:**
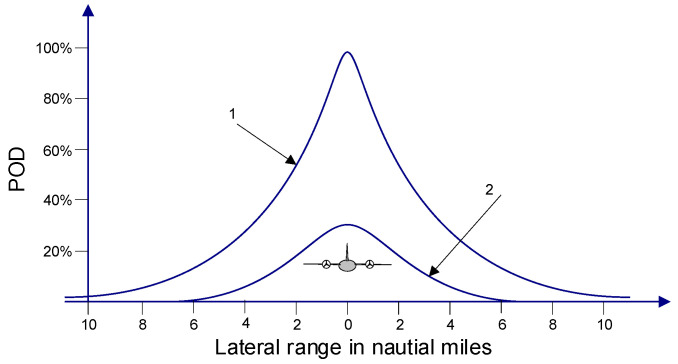
Probability of detection (POD)—visual search direction profiles for a single sweep width, search object: boat 7 m length, ideal search condition: 1—visibility 20 NM, calm weather, search attitude 300 m, corrected sweep width 5 NM, probable position error 0.25 NM; 2—visibility 20 NM, calm wind, search attitude 300 m, corrected sweep width 5 NM, probable position error 0.25 NM.

The probability of detection (POD) for the visual search of a boat for a single sweep width in different weather conditions is presented in Figure 25 [17].

**Figure 26:**
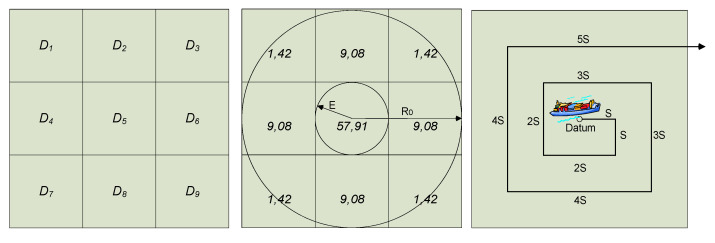
Search area divided into nine subareas. Expanding square search (SS) pattern.

The most effective search pattern defined as a track line assigned to a search procedure of a rescue unit for searching the specified area when the location of the search object is known with a good accuracy is the expanding square search (Figure 26). 

If we assume the determined search area is divided into subareas as in Figure 19, the condition for detecting an object in a designated area is that the object is in this area at the time of search. The total probability of the search object detection by a rescue vessel in one of the nine search subareas is given by Equation (7) [1].

(6) $$P(B_{j}(t))=\sum_{k=1}^{K}P(A_{k}(t))P(B_{j}(t)|A_{k}(t)),\;j=1,\;2,\ldots ,K,$$

where: P(A_k_(t)) is the probability that the search object is in the subarea A_k_ searched in time t; P(Bj(t)) is the probability that the search object is detected in time t.

The probability of detection (POD) assuming the search object is in the searched area is a function of a coverage factor, sensors characteristics, search conditions, and the accuracy of navigation along the assigned search pattern, and measures the sensor effectiveness under the prevailing search conditions [17].

Probability of detection (POD) is determined from Equation (7) [17].

(7) $$\mathrm{POD}=1-e^{-c},$$

where c = Z/A is the ratio of the search effort Z to the searched area A. The search effort Z is the measure of the area the rescue vessel can effectively search within the limits of search speed, endurance and sweep width. 

Search effort Z is a product of search speed V, search endurance T and sweep width W (8).

(8) Z=V⋅T⋅W

The probability of success POS (8) is the product of probability of detection POD and probability of containment POC—the probability that the search object is contained within the boundaries of the area [16].

(9) POS=POD⋅POC

**Figure 27:**
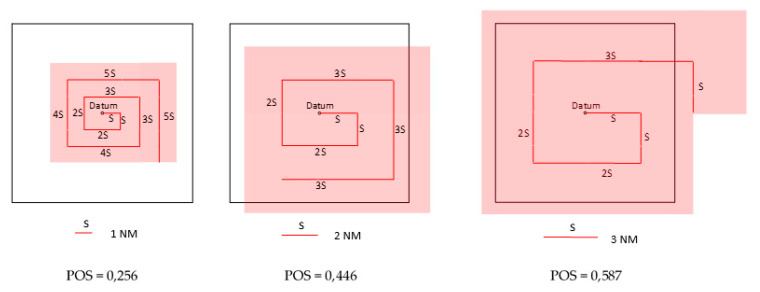
Expanding square search patterns. Probability of success for 1 NM, 2 NM and 3 NM sweep widths, S—the length of the side of expanding square.

The expanding square search patterns and probability of success for the assumed sweep widths W equal to 1, 2, 3 NM are presented in Figure 27.

The increase of the detectability distance allows to increase the search width W; and for W = 1–3 NM, the probability of detection is equal to 0.259–0.593 respectively. 

### 3.2. Influence of Detection Distance on the Probability of Success Related to the Survival Time of a Person in the Water

Probability of success when taking into account the survival time of a person in the water is expressed as a product of POC, POD and S_P_(t) (10):

(10) $$\mathrm{POS}=\mathrm{POC}\cdot \mathrm{POD}\cdot S_{P}(t),$$

Probability of detection and probability of success for different sweep widths and assumed probability of containment equal to 0.99 [16], calculated for the search endurance equal to 3 h and values of S_P_(t) for persons in the water at 5 °C, 10 °C, 15 °C, and 20 °C given in Figure 4, is presented in Table 1. 

For 1 h endurance time for PIW, the probability of survival is S_p_(t) ≈ 0.85 (Figure 4). This high value decreases significantly in time. The higher probability of detection increases the probability of success to 0.256–0.587; however, in low temperature, even the determined twice increase of POS is insufficient.

**Figure 28:**
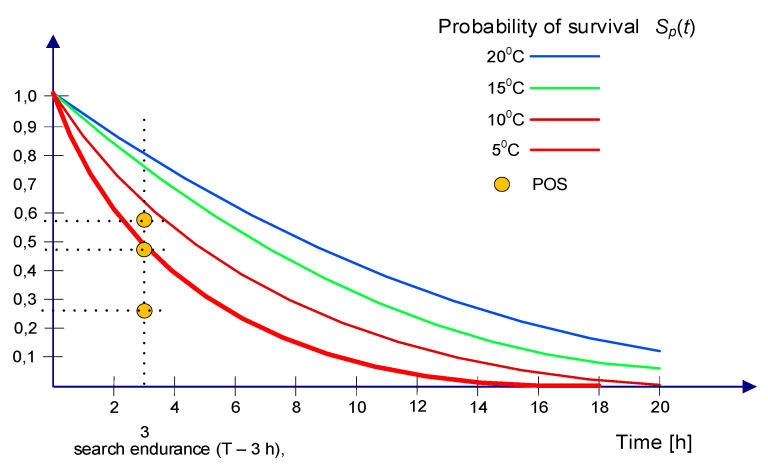
The influence of search endurance on the probability of survival of a person in the water related to the probability of success.

Figure 28 presents the influence of search endurance on the probability of success related to the survival time of a person in the water, which depended on water temperature for the assumed 3 h SAR action.

**Figure 29:**
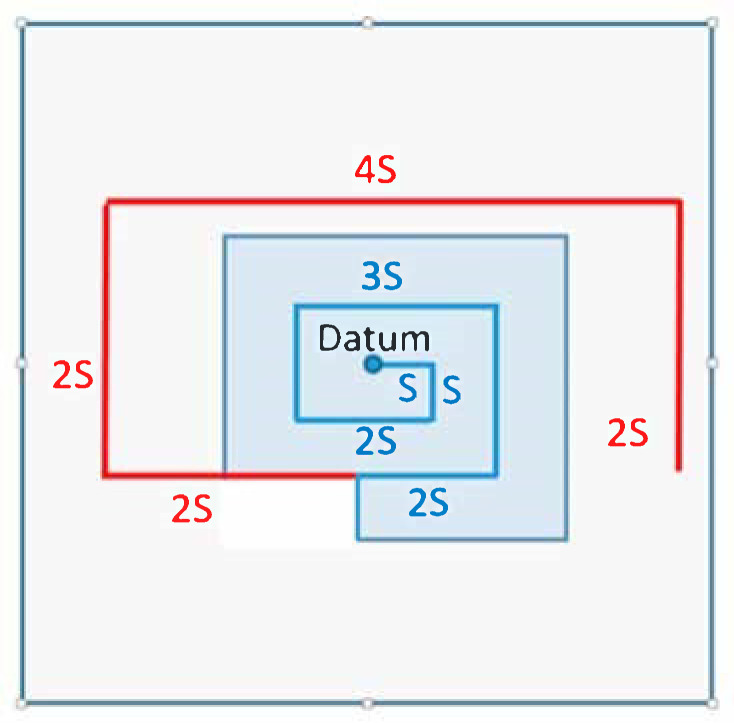
Search area of 100 NM^2^—the search pattern for the area of the highest probability of success marked in blue.

The width of the search path W recommended for PIW by IAMSAR is equal to 0.4 NM for 3 NM visibility and 0.7 NM for 20 NM visibility. The leeway for PIW is 0.25 to 0.6 knots for wind speed 34 knots [16]. If we assume W = 3 NM for a search object: PIW or life raft, they cannot be detected and not found, therefore, the smaller value of W = 0.7 NM should be considered during the first hour of search. The search pattern for the area of the highest probability of success presented in Figure 29 is marked in blue. 

For the search area of 100 NM^2^ determined for PIW, S should be equal to 0,7 NM during the first hour of search, then W can be widened to 2 NM for the life rafts and other search objects.

## 4. Conclusions

The tested materials have good emission properties in the thermal range. The presented summary showed that at 12 V a similar average temperature 31–33 °C was obtained for all tested mats. The tested mats were not implemented in the life raft prototype because of the weight of material and its necessary powering.

The conducted research showed that the most radar radiation energy is reflected by fabric No. 1 technical one-sided metallized ST55-110/21 polyester film combined with fabric. This material was implemented in the prototype construction.

The detection distance of the searched object on the water equipped with heating elements may increase up to three times compared to an object without them. The three to four times greater radiation energy reflected by the canopy made of metallized polyester film laminated to fabric increases the radar detection distance by about 12%.

The three times greater search effort, which is the product of search speed, search endurance and sweep width W, and is dependent on the detection distance, gives about twice greater probability of success of SAR action.

This paper did not consider other methods of decreasing the radar detection distance as, for example, a method based on the harmonic radar principle, which transmits at one frequency (S-band) and receives signals from a transponder located on the life raft, which doubles the frequencies which are received by another antenna working at C-band [24]. The future perspective is to use different methods to increase probability of search object detection e.g., improve leeway modelling and prediction and further improve construction materials characteristics and detection methods.

## Figures and Tables

**Table 1:** Probability of detection and probability of success for different sweep widths for probability of containment equal to 0.99.

W [NM]	C	Z	POD	POS	POS(PIW)			
					5 °C	10 °C	15 °C	20 °C
1	0.3	30	0.259	0.256	0.122	0.157	0.195	0.205
2	0.6	60	0.451	0.446	0.214	0.276	0.344	0.362
3	0.9	90	0.593	0.587	0.282	0.363	0.452	0.475
